# Nd:YAG capsulotomy incidence associated with five different single-piece monofocal intraocular lenses: a 3-year Spanish real-world evidence study of 8293 eyes

**DOI:** 10.1038/s41433-021-01828-z

**Published:** 2021-11-11

**Authors:** José I. Belda, Javier Placeres Dabán, Juan Carlos Elvira, Derek O’Boyle, Xavier Puig, Caridad Pérez-Vives, Ming Zou, Shaohui Sun

**Affiliations:** 1 Hospital Universitario de Torrevieja, Torrevieja, Alicante Spain; 2 Hospital Universitario del Vinalopó, Elche, Alicante Spain; 3 Alcon Laboratories Ireland Ltd., Cork, Ireland; 4 Alcon Healthcare, SA, Barcelona, Spain; 5 Alcon Management SA, Cointrin, Switzerland; 6 IQVIA Real World Solutions, Basel, Switzerland

**Keywords:** Implants, Technology

## Abstract

**Objectives:**

To investigate the associations between different single-piece monofocal intraocular lenses (IOLs) and neodymium-doped yttrium aluminum garnet laser (Nd:YAG) capsulotomy incidence 3 years after cataract surgery in a Spanish cohort.

**Methods:**

This is a longitudinal retrospective cohort study. Data were extracted from the electronic medical records of two large regional hospitals in Spain. Patients aged ≥65 years receiving cataract surgery with placement of five different IOLs and with ≥6 months of baseline data were included. We report the Nd:YAG capsulotomy incidence 3 years post cataract surgery, and the survival plot over the 3 years of follow-up time. The associated adjusted (age, gender, and diabetic retinopathy) multivariate analysis with odds ratios (ORs) and 95% CIs is also presented.

**Results:**

The cohort (53% female, mean age 75 ± 5.9 years) included 14,519 eyes (Alcon AcrySof = 2968, AJL LLASY60 = 1776, Medicontur Bi-flex = 5176, Zeiss Asphina = 4478, and IOL Tech Stabibag = 121). Of these, 8293 were retained until 3-year follow-up. At 3 years after cataract surgery, the Nd:YAG capsulotomy incidence was 5% for Alcon AcrySof, while it ranged from 21.2% to 31.1% for the other IOLs (*p* < 0.0001 for each comparison). The odds for Nd:YAG capsulotomy were significantly higher (*p* < 0.0001) for other IOLs compared with those of Alcon AcrySof (ORs = 8.85, 5.86, 5.74, 5.21 for AJL LLASY60, Medicontur Bi-flex, IOL Tech Stabibag, and Zeiss Asphina, respectively).

**Conclusions:**

The lower Nd:YAG capsulotomy rates for Alcon AcrySof IOLs compared to the other IOLs support the importance of lens choice in reducing patient burden and treatment costs.

## Introduction

Cataracts are the leading cause of partial and complete blindness, accounting for approximately half of the blindness globally [1, 2]. Cataract surgery is one of the most common and successful procedures worldwide, given its high success rates in improving visual acuity [3, 4]. However, the procedure can also lead to posterior capsule opacification (PCO), a complication that can result in reduced visual acuity, impaired contrast sensitivity, and glare disability [5]. A recent Cochrane review reported PCO incidence rates of up to 43% within the first year after cataract surgery [6]. The standard treatment for the post-surgery PCO complication is neodymium-doped yttrium aluminum garnet (Nd:YAG) laser capsulotomy [5]. While the treatment is generally considered safe, it poses additional treatment burden to patients and is associated with a number of complications, which can increase the overall healthcare costs [5, 7]. Thus, to optimize the patient outcomes and costs, PCO risk reduction is of utmost importance in terms of the current cataract surgical practice and also from the perspective of resource-constrained healthcare systems. The incidence of PCO has previously been associated with patient and procedure-related factors, such as age, ocular comorbidities, surgical technique, and IOL material and design [8, 9]. Much research effort has therefore focused on understanding the impact of potentially modifiable factors, including IOL material and design, on PCO (for a detailed review, see [10]). Previous research assessing the incidence of Nd:YAG capsulotomy in patients with different types of IOLs has reported more favorable results for hydrophobic IOLs compared to those made from other materials, including hydrophilic and PMMA, possibly due to their superior bioactivity [8, 9, 11–13]. While recent work from Scandinavia and the UK showed that AcrySof hydrophobic IOLs were associated with lower PCO incidence and Nd:YAG capsulotomy compared to certain hydrophobic and hydrophilic acrylic IOLs within 3–5 years following lens replacement. Head-to-head research assessing hydrophobic and hydrophilic IOLs is frequently reported in the literature. However, there is a paucity of comparative research when it comes to IOLs that have hydrophilic with hydrophobic surface properties, such as the Zeiss Asphina lens.

The current study provides comparative evidence for less frequently studied IOLs by offering new insights into the long-term Nd:YAG capsulotomy rates associated with five different IOLs (Alcon AcrySof, AJL LLASY60, Medicontur Bi-flex, IOL Tech Stabibag, and Zeiss Asphina) from a Spanish real-world perspective at 3-years post cataract surgery.

## Methods

### Study design and data source

This retrospective observational cohort study used the routine clinical data of patients who underwent cataract surgery. Their data were captured in electronic medical records (EMR) that were included in the Ribera Salud’s proprietary (Florence) database. The extracted data comprise records from two large Spanish regional hospitals of the Ribera Salud group in the Torrevieja-Vinalopó healthcare area that are main providers of ophthalmic procedures in the Alicante region. The EMR covers different medical conditions in ophthalmology, including glaucoma, cataracts, retinal detachment, and contain data from over 20,000 cataract patients dating back to 2006. Patient retrospective data are available post-operatively through a unique patient identifier number. The data were fully anonymized and compliant with the Spanish data protection rules governing use of patient-level healthcare data, including anonymization of physician names in the dataset (as defined in the newly enforced EU General Data Protection Regulation, 25 May 2018). The study received ethical approval from Ribera Salud’s institutional ethics review board.

### Study population

**Fig. 1:**
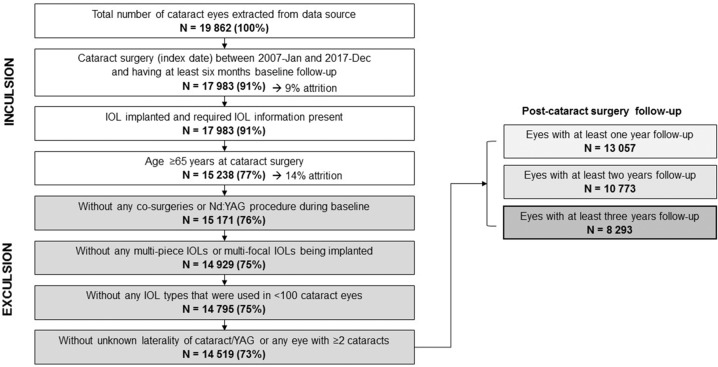
Population selection and attrition for eyes included in the study.

The study period comprised November 2006 to June 2019, capturing the most recent years of available data and variation in surgical procedures over time. The index date was defined at eye level as the date of cataract surgery for each eye operated between January 2007 and December 2017 (index period). All eligible eyes were followed up for 3 years (depending on the data availability) from the index to monitor Nd:YAG capsulotomy events. In case of a Nd:YAG capsulotomy event, the eyes were followed for up to 6 months to assess subsequent complications. Individuals were included if they had a record of cataract surgery to at least one eye within the index period; at least 6 months of available baseline data before surgery; in-the-bag placement of a single-piece, monofocal, acrylic IOL, available information on the IOL (i.e., material and manufacturer); and age ≥65 at cataract surgery (to ensure the inclusion of age-related cataracts). The exclusion criteria were any co-surgeries or Nd:YAG capsulotomy within 6 months before or during cataract surgery; implanted IOL that was used in <100 surgeries for the initial cohort; unknown eye laterality of cataract surgery or Nd:YAG capsulotomy procedure; and further cataract surgery on the same eye. Sample attrition is shown in Fig. 1. The initial cohort after applying the inclusion and exclusion criteria comprised 14,519 eyes with IOLs implanted during a cataract surgery (see Table 1 for included IOLs), of which 3 years' follow-up was completed for 8293 eyes (Alcon AcrySof = 1494 (hydrophobic), AJL LLASY = 1479, IOL Tech Stabibag = 95 (121 eyes in the initial cohort), Medicontur Bi-flex = 1520 (all hydrophilic), and 3777 Zeiss Asphina (hydrophilic with a hydrophobic surface)).

**Table 1:** Included IOL models.

IOL model	Optic concept	Material type	Optic edge design	Haptic design	*N*	%
Alcon AcrySof AU00T0	Single-piece, monofocal	Hydrophobic	Square edge (non-360°)	Stableforce modified L	452	3.1
Alcon AcrySof SN60WF	Single-piece, monofocal	Hydrophobic	Square edge (non-360°)	Stableforce	1359	9.4
Alcon AcrySof SN6AT^a^	Single-piece, monofocal	Hydrophobic	Square edge (non-360°)	Stableforce modified L	58	0.4
Alcon AcrySof other	Single-piece, monofocal	Hydrophobic	Square edge (non-360°)	Stableforce	1099	7.6
AJL LLASY60	Single-piece, monofocal	Hydrophilic	Square edge (360°)	Plate	1776	12.2
IOL Tech Stabibag	Single-piece, monofocal	Hydrophilic	Round	Tri-haptic	121	0.8
Medicontur Bi-Flex	Single-piece, monofocal	Hydrophilic	Square edge (360°)	C-loop	5176	35.7
Zeiss Asphina	Single-piece, monofocal	Hydrophilic with hydrophobic surface	Square edge (non-360°)	4-haptic. Microincision cataract surgery (MICS)	4478	30.8

^a^This model was used on ≥100 eyes but cases were excluded due to other exclusion criteria.

### Data extraction

The following variables were extracted from the EMR: gender, age at cataract surgery, number of eyes operated, year of cataract surgery, ocular co-pathologies (including diabetic retinopathy, glaucoma, uveitis, high myopia, and retinal detachment), IOL manufacturer/brand (see Table 1), Nd:YAG capsulotomy, and intra-/post-Nd:YAG capsulotomy procedure complications. Data were coded using system-wide Ribera Salud proprietary procedure codes, along with procedure descriptors and International Classification of Disease-10 diagnosis codes [14]. Natural language processing technology was used to capture the events and to assign the event to the correct eye; see [15, 16] for details on this methodology.

### Statistical analysis

All analyses were performed using SAS software version 9.4. Characteristics for the eyes included in the study were presented with the total number and percentage for categorical variables, and number, mean (standard deviation (SD)), median (interquartile range (IQR)), minimum, and maximum for numerical variables. Included eyes were stratified into five groups according to the different single-piece monofocal IOLs implanted at the study sites: Alcon AcrySof, AJL LLASY60, Medicontur Bi-flex, Zeiss Asphina, and IOL Tech Stabibag. For each group, records of Nd:YAG capsulotomy procedure incidence were evaluated over 3 years from cataract surgery (index) and 95% CI and *p*-values were calculated. The Bonferroni method was used to adjust for multiplicity; four comparisons were carried out therefore, each comparison was conducted at a level of significance of 0.0125. Survival curves were plotted using the Kaplan–Meier method to describe Nd:YAG capsulotomy incidence over the study time period (3 years), whereby the failure event was a record of Nd:YAG capsulotomy procedure.

In addition, adjusted odds ratios (ORs) for receiving Nd:YAG capsulotomy during the 3-year follow-up period were also computed. The adjusted model focused on the specific IOLs implanted and was adjusted for age at index, gender, number of eyes operated, diabetic retinopathy, glaucoma, uveitis, high myopia, and retinal detachment, which were selected with a step-wise approach where a significance level of 0.2 was required to allow a variable into the model, and a significance level of 0.1 was required for a variable to stay in the model.

## Results

### Baseline demographics and characteristics of the selected population

Patient baseline demographic and characteristics are presented in Table 2. A total of 14,519 eyes from 9545 patients were included in the analysis. The overall sample was aged 75 ± 5.9 years at cataract surgery, with a comparable mean age across IOL groups, ranging from 74.9 years for the AJL LLASY60 cohort to 75.5 years for the IOL Tech Stabibag cohort. Forty-eight percent of the patients had surgery on a single eye and 53% were female. Sample and eye-level characteristics, including eyes operated for cataract, were similar among IOLs, apart from the IOL Tech Stabibag, which had a higher proportion (92%) of patients with a single eye operated. The co-pathologies within 6 months before or during cataract surgery were also similarly distributed among the different IOLs, with glaucoma being the most common co-pathology (3.9% of all eyes, 3.3% for Alcon AcrySof, and 2.5% to 5.8% for the other IOLs).

**Table 2:** Baseline demographic and eye-level characteristics.

Demographics and characteristics	Overall		Alcon AcrySof		AJL LLASY60		Medicontur Bi-Flex		Zeiss Asphina		IOL Tech Stabibag	
	*N*	%	*N*	%	*N*	%	*N*	%	*N*	%	*N*	%
Patient-level characteristics												
Number of patients with cataract surgery and implanted IOLs	9545		1931		1221		3318		2966		109	
Patients with single or both eyes operated^b^												
Single eye operated	4571	47.9%	933	48.3%	666	54.5%	1435	43.2%	1437	48.4%	100	91.7%
Both eyes operated	4974	52.1%	998	51.7%	555	45.5%	1883	56.8%	1529	51.6%	9	8.3%
Gender												
Female	5085	53.3%	1049	54.3%	622	50.9%	1720	51.8%	1627	54.9%	67	61.5%
Male	4460	46.7%	882	45.7%	599	49.1%	1598	48.2%	1339	45.1%	42	38.5%
Eye-level characteristics												
Number of eyes with cataract surgery and implanted IOLs	14,519		2968		1776		5176		4478		121	
Age at cataract surgery (years)												
Mean (SD)	75.1 (5.9)		75.4 (6.2)		74.9 (5.6)		75.3 (5.8)		74.9 (5.9)		75.5 (5.2)	
Median (IQR)	75 (70–79)		75 (70–80)		75 (70–79)		75 (71–80)		75 (70–79)		75 (72–78)	
Range (Min, Max)	(65, 99)		(65, 96)		(65, 94)		(65, 99)		(65, 97)		(65, 97)	
Co-pathologies within 6 months before or during cataract surgery												
Diabetic retinopathy	114	0.8%	37	1.2%	10	0.6%	33	0.6%	33	0.7%	1	0.8%
Glaucoma	569	3.9%	97	3.3%	74	4.2%	136	2.6%	259	5.8%	3	2.5%
Uveitis (iridocyclitis)	16	0.1%	4	0.1%	3	0.2%	4	0.1%	5	0.1%	0	0.0%
High myopia	58	0.4%	5	0.2%	14	0.8%	19	0.4%	20	0.4%	0	0.0%
Retinal detachment	33	0.2%	10	0.3%	2	0.1%	6	0.1%	15	0.3%	0	0.0%

^a^Note that one eye might have had more than one co-pathology.

^b^Proportion of patients undergoing cataract surgery on one or two eyes during the study period.

### Three-year incidence of Nd:YAG capsulotomy

**Fig. 2:**
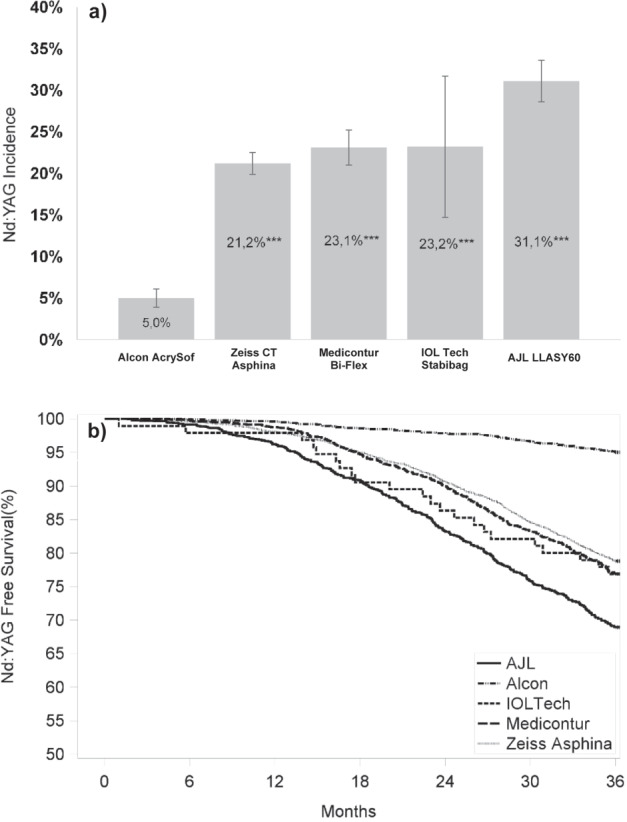
Nd:YAG capsulotomy incidence for the studied IOLs. **a** Nd:YAG procedure incidence proportions for different IOLs at 3-year follow-up. **b** Kaplan–Meier survival plots of eyes implanted with the five different IOLs. ***P < 0.0001 for each comparison with AcrySof.

The incidence proportion of Nd:YAG was 5.0% at 3 years after cataract surgery for Alcon AcrySof, while it ranged from 21.2% to 31.1% for the other IOLs (*P* < 0.0001 for each comparison) (Fig. 2a). The survival rate of Alcon AcrySof IOLs was consistently the highest compared to other IOLs over the follow-up period, post cataract surgery. Moreover, the difference in survival rate of Nd:YAG between Alcon AcrySof and other IOLs increased over the follow-up time, as illustrated by the Kaplan–Meier survival plot (Fig. 2b).

### Adjusted odd ratios of Nd:YAG capsulotomy

Multivariate analysis results with odds ratios (OR) adjusted for age, gender, and implanted IOL are shown in Table 3. Three years after cataract surgery, the ORs of Nd: YAG capsulotomy were at least 5.2 when comparing patients receiving non-Alcon IOLs with those receiving Alcon IOLs (ORs = 8.85, 5.86, 5.74, 5.21 for AJL LLASY60, Medicontur Bi-flex, IOL Tech Stabibag, and Zeiss Asphina, respectively, and *p* < 0.0001 for each pairwise comparison). AJL LLASY60 IOLs were associated with approximate nine-fold increased odds of Nd:YAG compared to Alcon AcrySof (adjusted OR: 8.85, 95% CI 6.82–11.47, *p* < 0.0001).

**Table 3:** Multivariate analysis showing adjusted ORs of Nd:YAG capsulotomy 3-years post cataract surgery.

Covariate^a^	Sub-category	OR (95% CI)	*p*-value
Age at index (per 1 year increase)		0.98 (0.97, 0.99)	<0.0001
Gender (Reference: male)	Female	1.28 (1.15, 1.44)	<0.0001
Number of eyes operated (Reference: 1)	2	–	–
	AJL LLASY60	8.85 (6.82, 11.47)	<0.0001
IOL brand (Reference: Alcon AcrySof)	Medicontur Bi-Flex	5.86 (4.50, 7.62)	<0.0001
	Zeiss Asphina	5.21 (4.07, 6.66)	<0.0001
	IOL Tech Stabibag	5.74 (3.37, 9.78)	<0.0001
Co-pathologies recorded prior to or on the index date (Reference: no)			
Diabetic retinopathy	Yes	1.71 (0.95, 3.06)	0.0717
Glaucoma	Yes	–	–
Uveitis	Yes	–	–
High myopia	Yes	–	–
Retinal detachment	Yes	–	–

^a^A significance level of 0.2 was required to allow a variable into the model, and a significance level of 0.1 was required for a variable to stay in the model.

Younger age, female gender, and diabetic retinopathy were also independently associated with an increase in the adjusted odds of Nd:YAG capsulotomy.

## Discussion

To our knowledge, this study is the first large Spanish cohort RWE study (+8000 eyes) that investigated the incidence of Nd:YAG capsulotomy after cataract surgery with different single-piece, monofocal, acrylic IOLs over 3 years. This research also serves to fill the gap with respect to infrequently studied IOLs in the PCO literature; in a recent systematic review on Nd:YAG capsulotomy [9], out of the 65 IOLs assessed in 67 publications, it was just the Alcon AcrySof lens that was present from the cohort of lenses assessed in this study. The overall incidence of Nd:YAG capsulotomy 3 years after cataract surgery was approximately 14%, and the rates were significantly lower for Alcon AcrySof IOLs (5%) compared to the other IOLs implanted at the study sites (31.1% for AJL LLASY60, 23.2% for IOL Tech Stabibag, 23.1% for Medicontur Bi-flex, and 21.2% for Zeiss Asphina, Fig. 2a) (*P* < 0.0001 for each comparison). The incidence of Nd:YAG capsulotomy increased from year 1 to year 3 post surgery, but was consistently about 80% lower for Alcon AcrySof compared to the other included IOLs. All non-Alcon IOLs were associated with over five-fold higher odds for Nd:YAG capsulotomy compared to Alcon AcrySof. At IOL level, Nd:YAG capsulotomy rates were highest for AJL LLASY60, followed by Medicontur Bi-flex, IOL Tech Stabibag (all hydrophilic), and Zeiss Asphina (hydrophilic with hydrophobic surface), and the lowest rates for Alcon AcrySof (hydrophobic) IOLs (Fig. 2a). Differences in Nd:YAG capsulotomy rates between the IOLs became more pronounced from 1 year to 3 years post cataract surgery. Our findings suggest a protective effect provided by Alcon AcrySof IOLs compared with other IOLs. This effect could be due to the specific material characteristics [10], as suggested by previous RWE studies, which showed lower PCO and Nd:YAG capsulotomy rates for hydrophobic compared with hydrophilic IOLs, with consistently superior outcomes for hydrophobic AcrySof IOLs compared to other IOLs [17–22]. The Nd:YAG capsulotomy rates in the current study were comparable to previous studies for the Alcon AcrySof IOLs (5% vs. 2.4% [21] and 3.9%), but more pronounced differences were present for the included IOLs that had hydrophilic surface characteristics (26.8% vs. 10.9% [21]). For IOLs with hydrophilic materials, we found the highest Nd:YAG capsulotomy incidence of 31.1% for AJL LLASY60 IOLs and the lowest for Medicontur Bi-flex and IOL Tech Stabibag (both ~23%). In comparison, the hydrophilic Zeiss Asphina IOLs with hydrophobic surface characteristics showed only a slightly lower Nd:YAG rate (21.2%), which was comparable to recent reports of Nd:YAG 3 years after cataract surgery by others (24.6%).

Our results indicate that the hydrophobic surface material is not as effective in preventing PCO as an entirely hydrophobic lens. Hydrophobic acrylic materials have a low water content and high fibronectin bio-adhesion properties. This bioactivity is thought to result in a sandwich pattern of adhesion between a single layer of lens epithelial cells, the posterior capsule, and the IOL body, thus allowing for better binding compared to other IOL materials, which could protect against PCO development [23, 24]. Possibly, the prevailing high water content (25%) may account for inferior fibronectin binding, while the existence of a rounder edge design may also play a role [25]. However, it is possible that other lens characteristics might account for the differences in performance with respect to Nd:YAG between IOLs. For instance, it has been suggested that the ‘stepped’ sharp edge, which extends beneath the uninterrupted broad haptic junction of the IOLs, may delay, but cannot prevent the occurrence of PCO. A recent comparison of PCO in square edged IOLs has reported more beneficial outcomes for IOLs with a radius of curvature of less than 10 μm, while the radius of curvature of Asphina is 13.7 μm. In contrast, and although both the Zeiss Asphina and AcrySof models have a similar edge design (square edge, non-360°), the AcrySof lens has a radius of curvature (8.5μm) smaller than 10 μm, which may offer a further rationale as to why AcrySof models have demonstrated lower PCO rates. Also, IOLs manufactured with sharp optic edges have been shown to be more effective in preventing PCO by creating a mechanical barrier at the capsular bend compared to round-edged IOLs. Compared with the hydrophobic IOLs, the high water content and specific manufacturing process of hydrophilic IOLs has been suggested to result in a rounder edge profile, which could account for higher rates of PCO and subsequent Nd:YAG [26]. Besides, differences in the Nd:YAG incidence rates by different IOLs in our study could also be due to other IOL properties, for instance, different lens size or haptic design characteristics between the stableforce vs. plate, tri-, or c-loop haptic designs of the hydrophilic IOLs, as previously suggested by other RWE studies [19]. The findings highlight the need for further research to fully understand the clinical consequences of IOL choice and material characteristics.

While the direction of the current findings was in line with previous work, the overall Nd:YAG capsulotomy incidence was substantially higher, which might be due to the specific sample characteristics [19, 21, 22]. Moreover, while the investigation of risk factors for Nd:YAG capsulotomy was not our main aim, the multivariate analysis showed that diabetic retinopathy, younger age at cataract surgery, and female gender were associated with higher risk for PCO, in line with the findings previously reported by others [21]. High myopia was associated with an increased risk of Nd:YAG capsulotomy in the univariate analysis (see Supplementary Material); however, this association did not persist in the adjusted multivariate analysis. Conflicting results have been reported with respect to whether myopia is related to PCO development [27, 28].

The average incidence of post-Nd:YAG capsulotomy complications within 6 months for the study cohort was 7.1% (95% CI 5.9–8.3); the most frequently observed complications were posterior vitreous detachment, macular atrophy, and cystoid macular oedema.

This study is the first to report on RWE evidence from Spain relating to the incidence of Nd:YAG capsulotomy associated with single-piece monofocal IOLs, showing significant differences in Nd:YAG rates between IOLs. The strengths of this study are the inclusion of a large sample that has been followed over 3 years post cataract surgery and the presentation of novel RWE insights on specific postoperative Nd:YAG capsulotomy treatment outcomes associated with different IOLs in a Spanish patient population. Another strength of our study is that the two hospitals that contributed data (Ribera Salud hospitals) are the hospitals in Spain that have fully adopted electronic medical record and resource management systems, which offered a unique opportunity to conduct this retrospective study using secondary data feasible in a Spanish setting. In addition, the Ribera Salud hospital network is a leading healthcare system using the capitation model in Spain. This model attracts patients to stay long within the care system and accumulates more longitudinal and complete follow-up data, which can be utilized by future RWE studies.

While our findings are important, they need to be interpreted in the light of the methodological consideration that the RWE data may over-represent more severe cases, given that these might be seen more frequently compared to cases with mild PCO or those who responded well to surgical treatment. A further limitation relates to potential biases that may arise from certain variables that may also play a role in PCO formation that were not controlled for in the conducted multivariate analysis. Examples of such factors from recent work include axial length, topical steroids, and nonsteroidal anti-inflammatory medications (NSAIDs) [29] and the dioptric power of the IOL implanted at the time of surgery [30]. Moreover, potential biases associated with between-eye correlation were not controlled for in this research; future work could consider these factors as part of the statistical analyses.

To conclude, the finding that Alcon AcrySof IOLs are associated with a lowest incidence of Nd:YAG capsulotomy compared to all other IOLs implanted at the study site has important clinical implications. Crucially, IOL choice may reduce patient burden and improve quality of life after cataract surgery. In addition, a reduced need for Nd:YAG capsulotomy after cataract surgery is likely to reduce clinical risks and subsequent healthcare costs. Further research is needed to investigate the relationship between specific IOL design characteristics and other confounding factors that may impact Nd:YAG capsulotomy rates and the related clinical risks and treatment costs associated with the procedure.

### Summary

#### What was known before

- Prior real-world evidence associated hydrophobic AcrySof IOLs with reduced rates of PCO and Nd:YAG capsulotomy compared to other materials and design of IOLs.

#### What this study adds

- This study extends previous insights to a Spanish cohort and shows that Alcon AcrySof IOLs are associated with lower Nd:YAG capsulotomy rates compared to other single-piece monofocal lenses, which include AJL LLASY60, Medicontur Bi-flex, and IOL Tech Stabibag, which are hydrophilic IOLs, and Zeiss Asphina, which are hydrophilic IOLs with hydrophobic surface.

## Supplementary information

Supplementary Material
