# Supplementary material for: Nd:YAG capsulotomy incidence associated with five different single-piece monofocal intraocular lenses: a 3-year Spanish real-world evidence study of 8293 eyes
Source: Eye (Lond). 2021 Nov 11;36(11):2205–10. doi: 10.1038/s41433-021-01828-z (PMC9581982; doi:10.1038/s41433-021-01828-z)
Supplement: Supplementary file 1 — Supplementary Material [file 41433_2021_1828_MOESM1_ESM.docx]

**Supplemental material.**

Univariate analysis showing unadjusted ORs of Nd:YAG capsulotomy 3-years post-cataract surgery

| **Covariate*** | **Sub-category** | **OR (95% CI)** | **p-Value** |
| --- | --- | --- | --- |
| Age at index (per 1 year increase) |  | 0.98 (0.97, 0.99) | <.0001 |
| Gender (Reference: Male) | Female | 1.23 (1.10, 1.37) | <.0001 |
| Number of eyes operated (Reference: 1) | 2 | 0.98 (0.87, 1.10) | 0.743 |
|  |  |  |  |
|  | AJL LLASY60 | 8.64 (6.67, 11.21) | <.0001 |
| IOL brand (Reference: Alcon AcrySof) | Medicontur Bi-Flex | 5.76 (4.43, 7.49) | <.0001 |
|  | Zeiss Asphina | 5.16 (4.04, 6.61) | <.0001 |
|  | IOLTech Stabibag | 5.78 (3.40, 9.83) | <.0001 |
|  |  |  |  |
| Copathologies recorded prior to or on index date (Reference: No) |  |  |  |
| Diabetic retinopathy | Yes | 1.45 (0.83, 2.54) | 0.1889 |
| Glaucoma | Yes | 1.21 (0.95, 1.55) | 0.1175 |
| Uveitis | Yes | 0.87 (0.19, 4.04) | 0.8602 |
| High myopia | Yes | 2.13 (1.08, 4.20) | 0.0283 |
| Retinal detachment | Yes | 0.62 (0.18, 2.09) | 0.441 |

* A significance level of 0.2 was required to allow a variable into the model, and a significance level of 0.1 was required for a variable to stay in the model
